# Teeth, size or screen time? How media and biological traits shape public interest in dinosaurs

**DOI:** 10.1098/rsbl.2026.0425

**Published:** 2026-11-11

**Authors:** Pâmela Lima, Sebastián M. Santiago, Mikaella R. Silva, Bárbara Cabral, Karoline Azevedo, Vitória França, Aline M. Ghilardi, Adam Kane, Grace Nolan, Ricardo A. Correia, Richard J. Ladle

**Affiliations:** ^1^ Institute of Biological and Health Sciences, Federal University of Alagoas, Maceió, Brazil; ^2^ Diversity, Ichnology and Osteohistology Laboratory (DInO-Lab), Department of Geology, Universidade Federal do Rio Grande do Norte Natal, Rio Grande do Norte, Brazil; ^3^ Paleontology Sector, Câmara Cascudo Museum, Universidade Federal do Rio Grande do Norte, Natal, Rio Grande do Norte, Brazil; ^4^ School of Biology and Environmental Science, University College Dublin, Dublin, Ireland; ^5^ Department of Biodiversity Sciences, University of Turku, Turku, Finland; ^6^ Helsinki Lab of Interdisciplinary Conservation Science (HELICS), Department of Geosciences and Geography, University of Helsinki Helsinki, Finland; ^7^ Helsinki Institute of Sustainability Science (HELSUS), University of Helsinki, Helsinki, Finland

**Keywords:** culturomics, public interest, social extinction

## Abstract

Dinosaurs provide a unique lens for examining how biological traits and cultural processes interact to shape public interest in biodiversity. Using a dataset of 323 valid genera and over 3.5 billion Wikipedia pageviews collected between 2015 and 2025, we applied culturomic methods to test whether attention is best explained by intrinsic characteristics (body size, diet and geological epoch) or extrinsic cultural amplification (taxonomic history and media exposure). Our models reveal that appearance in the *Jurassic Park* movie franchise is the strongest predictor of contemporary public interest, surpassing biological traits such as size or trophic level. Larger, faunivore genera described earlier, and especially those featured in the *Jurassic Park* franchise, attract disproportionate public interest, whereas herbivores, omnivores and Mid‑Jurassic taxa receive comparatively less attention. These findings demonstrate that cultural representation can sometimes override biological disadvantages, concentrating visibility on a narrow subset of charismatic taxa. Recognizing this ‘blockbuster effect’ provides lessons for contemporary conservation communication: by leveraging narrative-driven media, scientists and practitioners can generate cultural visibility and mitigate the decline of collective attention and memory for less charismatic species.

## 1. Introduction

Dinosaurs have a far more pervasive global cultural presence than any other extinct group [1]. Although the last non-avian dinosaur died over 66 million years ago, they are among the most widely recognized and culturally embedded organisms on Earth, acting as powerful artefacts that link personal childhood memories with the planetary past [2]. Dinosaurs are unique in the way they sit at the intersection of science, culture and imagination, from education to global entertainment industries. This raises a fundamental question: is their enduring appeal driven primarily by intrinsic biological features such as body size, diet and perceived danger or by the processes of cultural amplification that shape how these organisms are depicted and encountered? This question was of particular concern to the eminent palaeontologist Stephen Jay Gould when writing in 1993 about the release of the first *Jurassic Park* film. Gould [3] observed that the franchise represented palaeontology's ‘greatest opportunity and our most oppressive incubus’—a spur for unparalleled general interest in palaeontology, but also a commercial flood that risked turning dinosaurs from objects of wonder into clichés and commodities.

Gould [3] recognized that public interest was driven by the intrinsic features of dinosaur biology ‘big, fierce and extinct’, as he succinctly phrased it. Studies of extant taxa show that large-bodied species attract higher public attention owing to well-documented human preferences for megafauna [4,5], while carnivorous taxa may evoke heightened interest through associations with predation, threat and fear [6,7]. Notably, both physical size and perceived harmfulness have been identified as the primary drivers of societal interest across the tree of life [8]. Similarly, species from particular geological intervals may be more salient owing to evolutionarily-derived differences in diversity and morphology, or owing to perceived antiquity. These explanations suggest that patterns of public attention should be strongly rooted in the properties of organisms themselves, consistent with broader cognitive and perceptual biases in how humans engage with the natural world [9].

An alternative, but not mutually exclusive, explanation is that public interest in dinosaurs is shaped by cultural processes that amplify the visibility of particular taxa. Media representations, historical contingency and educational exposure may all contribute to the feedback loops in which a small number of species become disproportionately prominent [10]. Indeed, for extinct organisms, the distinction between biological and cultural drivers is inherently blurred, as intrinsic traits are almost exclusively encountered through cultural outlets [1,8]. While natural history museums provide the authentic material necessary to stimulate primary interest [11], for many global audiences, knowledge of dinosaur biology is heavily mediated entirely through secondary cultural representations [12]. This reinforces the role of media as the primary lens through which these species are ‘experienced’ [8,13].

The global success of the *Jurassic Park* franchise, for example, has repeatedly elevated specific dinosaur genera into the public spotlight, potentially decoupling patterns of interest and attention from underlying biological traits [1,12]. However, this contemporary ‘dinomania’ [3] builds upon a pre-existing foundation of universal popularity that predates the digital age, rooted in a long history of museum representation and cultural symbolism. The ‘zoological otherness’ is pointed as the possible aspect of fascination with dinosaurs, as creatures that remain perpetually out of reach [2,14]. This tension between familiarity and otherness keeps the dinosaur in ‘constant symbolic motion’ between scientific fact and cultural fantasy, where the desire to reconstruct extinct species often reflects an eagerness for human self-discovery [14].

Other extinct taxa demonstrate similar enduring salience without the influence of massive cinematic productions; for example, the dodo (*Raphus cucullatus*) remains one of the most viewed species in digital encyclopaedias, driven by its status as a premier icon of extinction and its deep integration into literature and language [15]. These examples suggest that public interest emerges from complex interactions where media franchises do not create fascination ex nihilo, but rather amplify existing symbolic values and historical legacies [15]. Such dynamics can now be quantified using culturomics, a field of study that seeks to understand human culture through the quantitative analysis of changes in word frequencies in large bodies of digital texts [16] and has been increasingly applied to human relationships with nature [17,18].

Here, we use culturomic data to disentangle the relative contributions of intrinsic biological characteristics and cultural amplification in shaping global public interest in dinosaurs. By integrating data on species traits, historical descriptions and media exposure, we test whether patterns of attention are better explained by what dinosaurs are or by how they are portrayed. In doing so, we aim to provide a quantitative framework for understanding how natural and cultural processes interact to structure public engagement with biodiversity. This is particularly relevant because public interest plays a central role in shaping scientific agendas, educational priorities and conservation outcomes. In addition, it may have broader socioeconomic and political impacts, influencing the museum visibility, heritage policies, scientific tourism and the capacity of particular countries or institutions to project their palaeontological heritage globally [19–22]. While this attention is often unevenly distributed [23,24], understanding the drivers of such imbalance, even in extinct taxa, is an important challenge for global conservation efforts that increasingly depend on public engagement [25]. Another relevant point is that the unequal distribution of attention given to extinct taxa may also reflect asymmetries in scientific and cultural influence, determining not only which organisms become iconic but also which fossil heritages, and whose, gain greater international visibility [22].

## 2. Material and methods

### (a) Creation of the dinosaur genus database

A dataset was compiled by extracting an initial list of 1860 potential dinosaur genera from Wikipedia [26]. Focusing the analysis at the genus rather than the species level minimizes taxonomic issues, such as misidentification and instability. This preliminary list was subjected to a filtering process to remove duplicate entries, invalid names and taxa that do not correspond to dinosaurs. Taxa of uncertain taxonomic status (e.g. *Dracorex* and *Troodon*) were retained given their widespread representation in popular culture. Following this stage, we used a custom Python script to check for corresponding pages on Wikipedia, as well as to retrieve their URLs and the respective associated QIDs (a persistent Wikidata identifier assigned to each entity, used to unambiguously reference and link data across systems). These QIDs provided stable, language-independent identifiers that enabled reliable cross-language linkage and automated data collection without relying on page titles alone [27].

Having established a cleaned base list of dinosaur genera, taxonomic classification (clade) and dietary information was gathered using the Paleobiology Database (PBDB) and manually curated [28]; dietary categories (e.g. herbivore, faunivore and omnivore) were assigned based on clade-level ecological consensus supported by specific literature references (full details in the supplementary material). To obtain standardized biological and historical attributes, including estimated body size (length in metres), year of description and geological epoch (Late Triassic, Early, Mid and Late Jurassic and Early and Late Cretaceous), data were retrieved from the Natural History Museum of London (NHM) dinosaur directory [29]. To ensure data consistency, minimize missing values and increase the reliability of the variables used in our statistical models, we retained only the genera that were present in the NHM dataset. This final filtering step yielded a dataset of 323 valid dinosaur genera.

### (b) Cultural variables: the *Jurassic Park* franchise

To test our hypothesis regarding the ‘blockbuster effect’ on the public visibility of certain dinosaur genera, the *Jurassic Park* franchise was chosen, as it is a cinematic phenomenon featured in cinemas all around the world, that spans generations through books, movies, video games and other pop culture and educational content [30,31]. Information on the presence of dinosaur genera in the franchise was obtained from a consolidated Wikipedia list of taxa featured across the films [32]. For each genus, we recorded: (i) whether it appeared in at least one film (presence/absence) and (ii) the year of its first appearance in the franchise. Only valid, non-fictional genera belonging to Dinosauria were included in this analysis.

### (c) Public interest proxy

Public interest was quantified using Wikipedia pageview statistics, a metric widely used as a proxy for public attention in culturomics research. Using a custom R script, we collected daily pageview data for each of the 323 genera across all available language editions of Wikipedia, identified via their corresponding QIDs. Data were obtained through the Wikipedia pageviews API and restricted to human users only (agent = user), explicitly excluding bots and automated traffic.

The time window spanned from 1 July 2015 to 1 July 2025, corresponding to the period for which standardized pageview data were available. For each genus and language, we calculated the average daily number of views by dividing the total number of views by the number of sample days available for that language. These language-specific daily averages were then aggregated across all available languages to generate a single digital public interest (DPI) metric for each genus.

We explicitly recognize that Wikipedia pageviews measure information-seeking behaviour rather than knowledge accuracy, attitudes or conservation support. Therefore, they are treated here as a proxy for visibility and attention. It should also be noted that Wikipedia pageviews capture only one dimension of public interest and that observed patterns may be influenced by language availability, editorial quality and internet access, and should be interpreted as indicators of attention rather than direct indicators of conservation support.

### (d) Statistical analyses

Summary statistics were compiled for each geological epoch, including sample sizes, mean public interest, body length and diet, to provide a descriptive overview of the dataset. To evaluate which biological, cultural and historical attributes best predict public interest in dinosaurs, a generalized linear model (GLM) with a Gaussian error distribution and identity link was fitted. DPI (log_10_-transformed DPI) served as the response variable. The model included three categorical predictors, appearance in *Jurassic Park*, diet and geological epoch, and two continuous predictors, body length and year of taxonomic description. Continuous predictors were mean-centred and divided by one standard deviation prior to model fitting to allow comparison of effect sizes across predictors. Multi-collinearity among predictors was evaluated by examining pairwise correlations among quantitative predictors and by calculating variance inflation factors (VIF) for all model terms. A chi-square test of independence was used to assess whether diet was associated with the probability of appearing in the *Jurassic Park* franchise. Model assumptions were validated using the DHARMa package in R [33]. All statistical analyses were conducted in R Software [34]. Statistical significance was evaluated at *α* = 0.05. See the supplementary material for further details on model specification and diagnostics.

## 3. Results

Descriptive statistics for dinosaur genera across geological epochs are provided in table 1. The distribution of DPI across these epochs, highlighting the most highly viewed genera, their relative body sizes and their appearance in the *Jurassic Park* franchise, is illustrated in figure 1.

**Table 1.** Summary statistics for dinosaur genera across geological epochs, including sample size, mean log_10_-transformed and untransformed DPI, proportion of genera featured in the *Jurassic Park* franchise, average body length (in metres) and proportion of faunivore genera (LT: Late Triassic; EJ: Early Jurassic; MJ: Mid Jurassic; LJ: Late Jurassic; EK: Early Cretaceous; LK: Late Cretaceous).

epoch	genera (*n*)	mean DPI (log_10_)	mean DPI	genera distribution (%) in JP	mean length size (m)	proportion of faunivore (%)
LT	15	4.08	50.1	0	3.69	40
EJ	20	1.60	99.36	5	5.51	20
MJ	26	3.51	31.2	0	8.18	33
LJ	43	4.65	87.6	16.2	9.80	34
EK	67	1.74	43.8	6	7.97	27
LK	150	4.32	53.9	13.3	6.78	22

**Figure 1.**
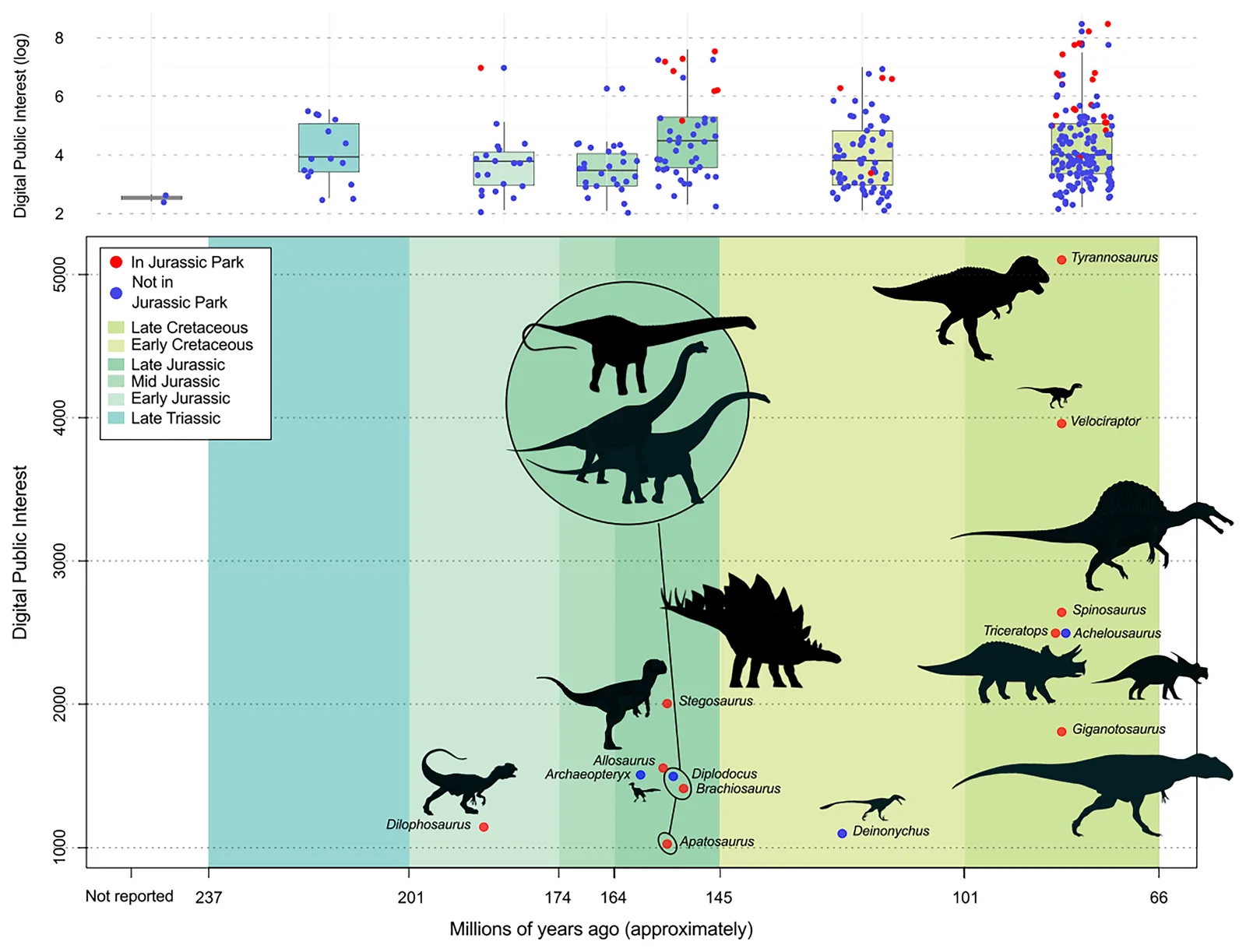
Average daily Wikipedia page views for dinosaur genera across geological epochs. Upper panel: log_10_-transformed values shown as boxplots with overlaid individual data points. Lower panel: untransformed values for the most visited genera (more than 1000), with illustrative silhouettes. Red and blue points indicate the presence or absence in the *Jurassic Park* franchise, respectively.

The generalized linear model explained a substantial proportion of the variation in DPI (adjusted *R*² = 0.46, *F* = 27.05, *p* < 0.001), with *Jurassic Park* appearance, body length, diet and year of description showing significant associations with DPI (supplementary material, table S1). Geological epochs showed limited effects, with only the Mid Jurassic differing significantly from the reference category (*β* = –0.28, *p* = 0.004). Appearance in the *Jurassic Park* franchise showed the strongest positive association with DPI (*β* = 0.86, *p* < 0.001). Body length was also positively associated with DPI (*β* = 0.10, *p* < 0.001). Herbivorous and omnivorous genera showed significantly lower interest than faunivore genera (herbivore *β* = –0.41, *p* < 0.001; omnivore *β* = –0.36, *p* < 0.001). Year of taxonomic description was negatively associated with DPI (*β* = –0.12, *p* < 0.001) (figure 2). Diet was not associated with the likelihood of appearing in the *Jurassic Park* franchise (Fisher's exact test, *p* = 0.153).

**Figure 2.**
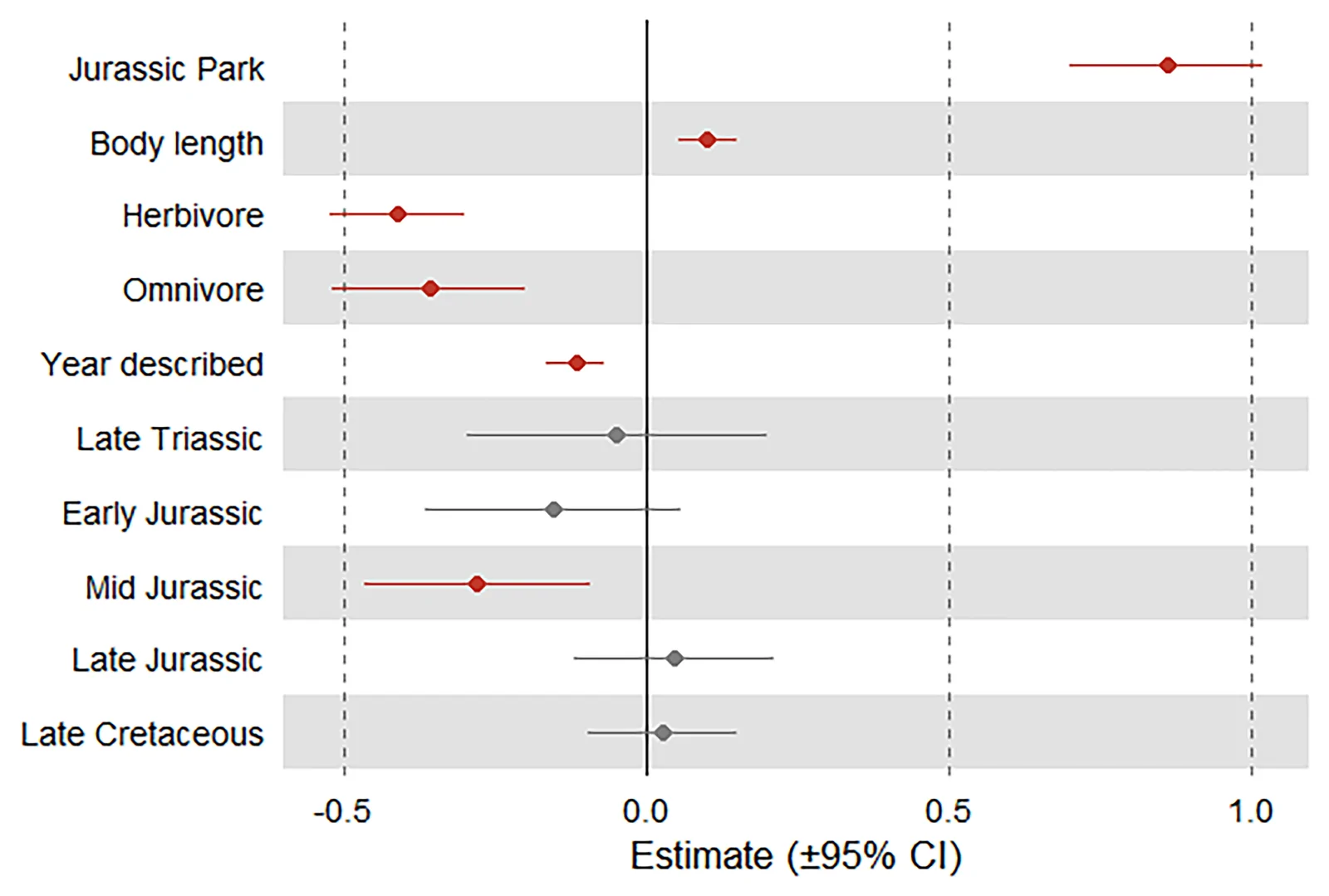
Forest plot showing the GLM coefficients for the associations between media exposure, historical attributes, biological traits and geological epoch on log_10_-transformed DPI in dinosaur genera. Points represent parameter estimates and horizontal lines indicate 95% confidence intervals. Point colour indicates statistical significance, with red points denoting significant effects (*p* < 0.05) and grey points indicate non-significant effects. Faunivore and Early Cretaceous were used as reference categories for diet and geological epoch, respectively. Because these categories are included in the model intercept, they are not shown separately in the figure.

## 4. Discussion

Our results suggest that cultural visibility of extinct species is not arbitrary but is strongly driven by a combination of media exposure, historical legacy and specific biological traits. By using dinosaurs as a model system, this study provides empirical evidence that public interest is disproportionately captured by large, predatory and historically well-established genera, a phenomenon further amplified by blockbuster media. Understanding the mechanisms that keep these extinct organisms ‘culturally alive’ offers insights into the challenge of societal extinction (the decline of collective attention and memory of a species) facing many extant species today [35].

Unsurprisingly, the strongest predictor of contemporary public interest in dinosaurs was appearance in the *Jurassic Park* franchise. This finding reinforces the premise that high-budget audio-visual media can be a powerful cultural anchor in shaping societal awareness and dictating which organisms remain relevant in the public imagination [13]. Importantly, several genera featured in the franchise, such as *Tyrannosaurus*, *Triceratops* and *Stegosaurus*, were already deeply embedded in popular culture through museum displays, early palaeo-art, children's books, films, advertising and world-fair exhibitions [36–38]. *Jurassic Park* did not create their cultural visibility, rather, it amplified and updated pre-existing palaeo-iconographic traditions [36,38]. Crucially, this influence extends beyond the initial surge of interest following the original 1993 film and Michael Crichton's novels; the sustained impact of subsequent instalments, including the *Jurassic World* trilogy, demonstrates how a long-running media franchise can continuously reinforce and update the cultural salience of specific taxa over several decades for many generations. Genera featured in the franchise command significantly more attention, demonstrating that media representation can effectively manufacture cultural visibility.

However, the effectiveness of this media representation relies on a pre-existing psychological and cultural foundation, with the public's enduring fascination with dinosaurs being fuelled by a unique intersection of nostalgia and ‘zoological otherness’ [2]. Dinosaurs act as intergenerational artefacts that bridge the temporal chasm between personal childhood memories and the deep planetary past, eliciting a powerful, hybrid sentiment of ‘dinostalgia’ [2]. It is precisely this tension between their familiar ubiquity in popular culture and their profound strangeness as extinct beings that makes them so alluring. However, this inherent capacity to spark fascination is not uniformly distributed across all dinosaurs.

Beyond media influence, intrinsic biological traits significantly bias public attention. Our model reveals a strong preference for large-bodied and carnivorous genera. These attributes probably tap into deep-seated narrative archetypes of power, danger and the sublime, suggesting that fear and fascination serve as drivers of public attention [39,40]. Much like the enduring cultural fascination with extant apex predators, such as sharks or big cats, large carnivorous dinosaurs evoke a primal emotional response that inherently attracts human attention [8]. Consequently, herbivorous and omnivorous species, despite their ecological dominance in the fossil record, remain culturally marginalized compared to their predatory counterparts.

The most profound implication of this study, however, lies in its translation to the conservation of extant biodiversity. The human preference for larger predators may have been one of the factors driving adaptations in the franchise; for example, the iconic *Velociraptor* in *Jurassic Park* was intentionally modelled after the much larger *Deinonychus* to appear more formidable on screen [41]. This pattern of interest can also be observed in the contemporary public's engagement with existing biodiversity. Research shows that human attention and conservation priorities disproportionately favour charismatic and large-bodied animals [42–46]. Specifically, large predators evoke a psychological mix of fear (biophobia) and admiration (biophilia) [46–48]. This ambivalence makes such animals more noticeable and memorable, ensuring greater social support for their protection, whereas smaller and less dangerous species tend to go unnoticed or be undervalued [46]. For example, modern apex predators, such as sharks, capture enormous cultural and media attention (exemplified by phenomena such as Shark Week), driven largely by their size and perceived danger [49], a privilege rarely extended to smaller taxa. If cultural relevance heavily gravitates towards these archetypes of large, fierce or media-endorsed organisms, the vast majority of living species—which are small, non-charismatic or culturally invisible—are at severe risk of societal extinction, which may precede and/or accelerate biological extinctions [35,50]. We cannot alter the biological traits of a threatened amphibian or insect to make them larger or more dangerous. We can, however, adapt the mechanisms that make dinosaurs culturally ubiquitous.

Alongside modern media and intrinsic biological biases, our model reveals that public interest is further compounded by historical momentum. We found that older taxonomic descriptions are associated with higher current public interest. Taxa described earlier have benefited from a ‘first-mover advantage’. They have had more than a century to permeate museums, literature and the popular imagination, cementing their place in the collective memory [51]. However, it is essential to contextualize these findings within the biases of the palaeontological record itself. Our analysis focuses on taxa that are already sufficiently well-preserved and scientifically described to possess robust data in the Natural History Museum directory. Therefore, our conclusions indicate that among dinosaurs with sufficiently complete fossil records, size, diet and media exposure drive public interest, rather than generalizing these drivers to all dinosaurian diversity. However, media representation can powerfully override these biological penalties; for instance, relatively smaller or non-carnivorous genera, such as *Triceratops* (2493 daily views), *Parasaurolophus* (955 daily views) and *Ankylosaurus* (945 daily views), achieved disproportionate cultural prominence almost entirely owing to their appearance in the movie franchise.

The primary contribution of these findings for biodiversity conservation lies in recognizing a critical reality—public fascination is often mediated not by direct interaction or ecological importance, but by compelling storytelling and cultural exposure. While digital proxies such as Wikipedia are sometimes critiqued for capturing only a fraction of total internet users (e.g. approx. 5000 daily views for *Tyrannosaurus*), analysing what drives this online interest and identifying the specific traits that attract public attention within a digital footprint reveals the immense power of cultural representation. Therefore, our findings suggest that conservation communication must move beyond merely presenting ecological facts. To counteract the cultural invisibility of non-charismatic species, conservationists must intentionally craft narrative archetypes and leverage modern media platforms. While fictional blockbusters like *Jurassic Park* can manufacture enduring cultural visibility for extinct taxa, non-fictional visual media has demonstrated a similar capacity to catalyse real-world social and policy changes for living biodiversity [52]. By giving living species compelling narratives, highlighting their unique evolutionary histories, bizarre behaviours or critical roles in human survival, we can simulate this ‘blockbuster effect’ on a smaller scale. Ultimately, if society can maintain a vibrant, collective fascination with animals that have been dead for 66 million years, there is an immense untapped potential to redirect that same cultural energy towards saving the species with which we still share the planet.

## 5. Conclusion

In conclusion, our study demonstrates that public engagement with extinct biodiversity is shaped by a predictable synergy between intrinsic biological characteristics and extrinsic cultural amplification. While physical size and predator status naturally attract human attention, extensive media coverage can reinforce or even override these biological signals. Crucially, these patterns provide a valuable roadmap for contemporary biodiversity conservation. By translating this ‘blockbuster effect’ into strategic narratives and narrative-driven communication, conservationists can intentionally create cultural visibility for smaller and lesser charismatic species, ultimately mitigating the growing threat of societal extinction.

## Supplementary Material



## Data Availability

Data are available at Zenodo [53]. Supplementary material is available online [54].
