# Supplementary material for: Teeth, size or screen time? How media and biological traits shape public interest in dinosaurs
Source: Biol Lett. 2026 Nov 11;22(11):20260425. doi: 10.1098/rsbl.2026.0425 (PMC13752743; doi:10.1098/rsbl.2026.0425)
Supplement: Supplementary file 1 [file rsbl.2026.0425.zip › rsbl.2026.0425/rsbl20260425_si_001.docx]

*Biology letters*

Supplementary Materials

**Teeth, Size, or Screen time? How media and biological traits shape public interest in dinosaurs**

Pâmela Lima^1*^, Sebastián M. Santiago^1*^, Mikaella R. S. Silva^1*^, Bárbara Cabral^1^, Karoline Azevedo^1^, Vitória França^1^, Aline M. Ghilardi^2,3^, Adam Kane^4^, Grace Nolan^4^, Ricardo A. Correia^5,6,7^ , Richard J. Ladle^1^

Affiliation:

^1^Institute of Biological and Health Sciences, Federal University of Alagoas, 57072-970, Maceió, Alagoas, Brazil.

²Diversity, Ichnology and Osteohistology Laboratory (DInO-Lab), Department of Geology, Federal University of Rio Grande do Norte, 59075-000, Natal, Rio Grande do Norte, Brazil.

³Paleontology Sector, Câmara Cascudo Museum, Federal University of Rio Grande do Norte, 59020-650, Natal, Rio Grande do Norte, Brazil.

^4^School of Biology and Environmental Science, University College Dublin, Dublin, Ireland.

^5^Department of Biodiversity Sciences, University of Turku, 20014 Turku, Finland.

^6^Helsinki Lab of Interdisciplinary Conservation Science (HELICS), Department of Geosciences and Geography, University of Helsinki, 00014 Helsinki, Finland.

^7^Helsinki Institute of Sustainability Science (HELSUS), University of Helsinki, 00014 Helsinki, Finland.

Corresponding author: pamela.lima@icbs.ufal.br

*Equal contributions

**Methods**

## **Creation of the Dinosaur Genus Database**

Taxa were assigned to specific dietary categories based on their phylogenetic affinities. Considering only non-avian dinosaurs, the following coding rules were applied to the dataset:

**Herbivores:** All taxa within Ornithischia, Sauropoda, and Therizinosauria were coded as herbivores.

**Herbivores-Omnivores:** Non-sauropod Sauropodomorpha were assigned to this category, with the exception of basal forms (including Guaibasauridae, Saturnaliidae, and the genera Eoraptor, Buriolestes, and Mbiresaurus), which were coded as uncertain. Additionally, Oviraptorosauria, Ornithomimosauria, and Confuciusornithidae were coded as herbivores-omnivores.

**Faunivores:** This category encompasses Herrerasauridae, Coelophysoidea, and most other theropod clades, including Dilophosauridae, Allosauroidea, Abelisauridae, Megalosauroidea, Megaraptora, Compsognathidae, Tyrannosauroidea, Dromaeosauridae, Alvarezsauroidea, and Scansoriopterygidae, as well as the genera Cryolophosaurus and Ceratosaurus. Any other theropod taxa not explicitly mentioned in these rules were also coded as faunivores by default.

**Faunivores-Omnivores:** Troodontidae and Anchiornithidae were coded under this category.

Noasauridae: Generally coded as faunivores, except for the clade Elaphrosaurinae and the genera Berthasaura and Afromimus, which were coded as herbivores-omnivores.

Because the dietary ecology of some taxa is debated, we applied a consistent operational criterion for the statistical analyses: taxa for which there was support for the consumption of both plant and animal resources were classified as omnivores. Thus, taxa assessed as herbivore-omnivore or faunivore-omnivore were included in the omnivore category. This approach was used to assign each taxon to a single trophic category, while acknowledging that the boundaries between faunivory and omnivory, and between herbivory and omnivory, are not always clear-cut.

**Table S1**: Results of a lineal model assessing the effects of appearance in the *Jurassic Park* franchise and biological traits on log₁₀-transformed daily Wikipedia interest. Estimates are shown with standard errors (SE), t values, and p values. Faunivore and Early Cretaceous (EK) were used as reference categories for diet and geological epoch, respectively. Statistical significance was evaluated at α = 0.05.

| **Predictor** | **Estimate (β)** | **SE** | **t value** | **p value** |
| --- | --- | --- | --- | --- |
| Intercept | 2.05 | 0.07 | 31.15 | < 0.001 |
| Jurassic Park appearance (JP = 1) | 0.86 | 0.08 | 10.63 | < 0.001 |
| Body length (m) | 0.10 | 0.03 | 4.06 | < 0.001 |
| Diet – Herbivore | −0.41 | 0.06 | −7.22 | < 0.001 |
| Diet – Omnivore | −0.36 | 0.08 | -4.41 | < 0.001 |
| Year of description | −0.12 | 0.02 | −4.71 | < 0.001 |
| Period - LT | -0.05 | 0.13 | -0.38 | 0.70 |
| Period - EJ | -0.15 | 0.11 | -1.43 | 0.15 |
| Period - MJ | -0.28 | 0.09 | -2.94 | 0.004 |
| Period - LJ | 0.04 | 0.08 | 0.55 | 0.58 |
| Period - LK | 0.03 | 0.06 | 0.42 | 0.68 |
